# Proinflammatory Mediators Enhance the Osteogenesis of Human Mesenchymal Stem Cells after Lineage Commitment

**DOI:** 10.1371/journal.pone.0132781

**Published:** 2015-07-15

**Authors:** Michiel Croes, F. Cumhur Oner, Moyo C. Kruyt, Taco J. Blokhuis, Okan Bastian, Wouter J. A. Dhert, Jacqueline Alblas

**Affiliations:** 1 Department of Orthopedics, University Medical Center Utrecht, Utrecht, the Netherlands; 2 Department of Surgery, University Medical Center Utrecht, Utrecht, the Netherlands; 3 Faculty of Veterinary Medicine, Utrecht University, Utrecht, the Netherlands; Ajou University, REPUBLIC OF KOREA

## Abstract

Several inflammatory processes underlie excessive bone formation, including chronic inflammation of the spine, acute infections, or periarticular ossifications after trauma. This suggests that local factors in these conditions have osteogenic properties. Mesenchymal stem cells (MSCs) and their differentiated progeny contribute to bone healing by synthesizing extracellular matrix and inducing mineralization. Due to the variation in experimental designs used *in vitro*, there is controversy about the osteogenic potential of proinflammatory factors on MSCs. Our goal was to determine the specific conditions allowing the pro-osteogenic effects of distinct inflammatory stimuli. Human bone marrow MSCs were exposed to tumor necrosis factor alpha (TNF-α) and lipopolysaccharide (LPS). Cells were cultured in growth medium or osteogenic differentiation medium. Alternatively, bone morphogenetic protein 2 (BMP-2) was used as osteogenic supplement to simulate the conditions *in vivo*. Alkaline phosphatase activity and calcium deposition were indicators of osteogenicity. To elucidate lineage commitment-dependent effects, MSCs were pre-differentiated prior treatment. Our results show that TNF-α and LPS do not affect the expression of osteogenic markers by MSCs in the absence of an osteogenic supplement. In osteogenic differentiation medium or together with BMP-2 however, these mediators highly stimulated their alkaline phosphatase activity and subsequent matrix mineralization. In pre-osteoblasts, matrix mineralization was significantly increased by these mediators, but irrespective of the culture conditions. Our study shows that inflammatory factors potently enhance the osteogenic capacity of MSCs. These properties may be harnessed in bone regenerative strategies. Importantly, the commitment of MSCs to the osteogenic lineage greatly enhances their responsiveness to inflammatory signals.

## Introduction

The autologous bone graft is currently considered the gold standard for bone repair and regeneration, but is associated with disadvantages such as limited availability and donor-site morbidity. Therefore, alternative approaches are being studied [1]. As a bone replacement therapy, osteoconductive materials lack the osteogenic and osteoinductive properties of autografts. They are therefore often combined with mesenchymal stem cells (MSCs) in an experimental setting [2,3]. Bone stimulating factors such as the bone morphogenetic proteins (BMPs) are also effective in enhancing bone regeneration, but it is uncertain if the delivery of BMPs alone induces the optimal pro-osteogenic environment required for bone regeneration [4,5]. To better mimic the physiological environment, a new approach is to identify the critical pro-osteogenic factors involved in inflammation and to harness them to promote bone formation in bone replacement strategies [6].

While uncontrolled inflammation often has destructive effects on bone [7], at the same time, there are numerous examples of how inflammatory processes trigger new bone formation. A classical example is found in the tightly controlled inflammatory phase after fracture, which initiates repair and is required for adequate bone remodeling [8,9]. Transgenic animal models have shown the pivotal role of tumor necrosis factor-alpha (TNF-α) and Interleukin-6 (IL-6) in fracture healing [10,11]. In line with these observations, pharmacological agents with an anti-inflammatory action impair proper healing [6,12]. Moreover, heterotopic ossifications and several diseases of the vertebral column associated with excessive bone formation are preceded by an inflammatory phase [13,14].

*In vitro*, inflammatory mediators control the proliferation, migration and differentiation of MSCs. TNF-α, IL-6 and IL-1β have been mainly studied due to their critical role in fracture repair [6,15]. The effects of these cytokines on MSC osteogenic differentiation have been contradictory [16–20]. As a strong activator of the immune system, the endotoxin lipopolysaccharide (LPS) has also been investigated. In MSCs, it induces cell migration and the production of several cytokines and chemokines [21]. On osteogenic differentiation, both stimulatory and inhibitory effects of LPS have been reported [22–25].

Due to the discordance in *in vitro* experimental designs used to study their osteogenic potential, there is still debate on whether proinflammatory mediators can be harnessed for bone regeneration strategies. The aim of the current study was therefore to assess the effects of the proinflammatory cytokine TNF-α and the endotoxin LPS on the osteogenicity of human bone progenitor cells. We sought to compare a number of parameters in parallel, i.e. the stage of lineage commitment, the timing of delivery, and the use of different osteogenic inducers. Under specific conditions, strong pro-osteogenic effects of these inflammatory stimuli were observed.

## Materials and Methods

### Experimental designs

Recombinant human tumor necrosis factor-alpha (TNF-α, 0.5–50 ng/mL; eBioscience, San Diego, CA, USA) and lipopolysaccharide (LPS, 0.05–5 μg/mL; from E. coli O55:B5, Sigma-Aldrich, St. Louis, MO, USA) were used as mediators. They were freshly added in the medium at each change.

**Fig 1:**
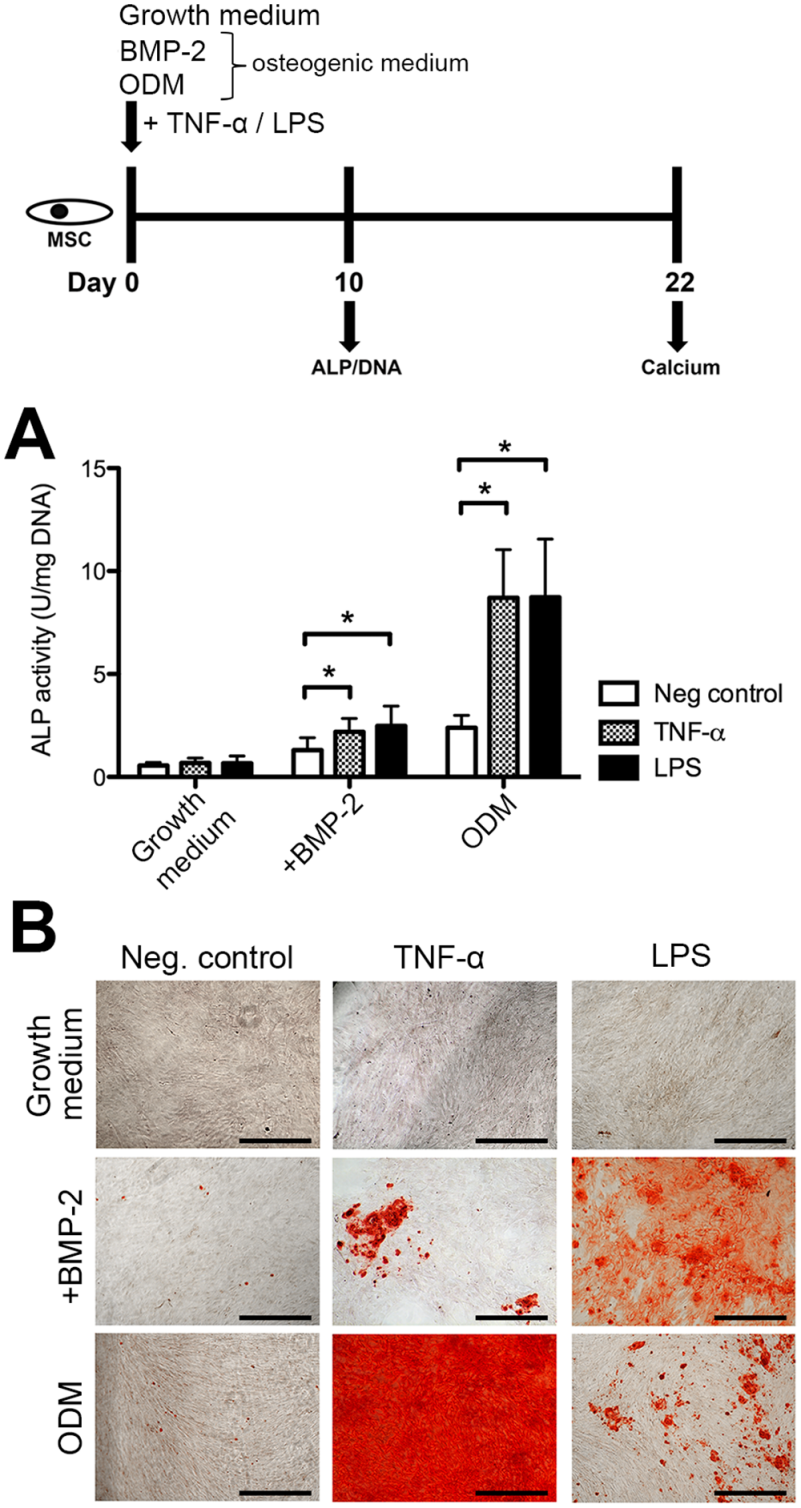
Expression of osteogenic markers by MSC treated with proinflammatory mediators. MSCs were exposed to TNF-α (5 ng/mL) or LPS (0.5 μg/mL). A. ALP normalized for DNA content was measured at day 10. Bars represent the means ± SD (*n* = 6). *P<0.05 compared to untreated cells cultured in the same medium. B. Alizarin Red S staining was performed to demonstrate matrix mineralization (representative for 4 donors). Scale bar: 500 μm. See S2A and S2B Fig for all concentrations.

In the first experimental design (Fig 1), MSCs were continuously exposed to TNF-α and LPS. To induce differentiation, osteogenic medium was used, while growth medium served as a negative control. Enzymatic alkaline phosphatase (ALP) activity was measured at day 10 as the levels of this marker generally peak around this time point [26]. Calcium deposition was analyzed at day 22.

**Fig 2:**
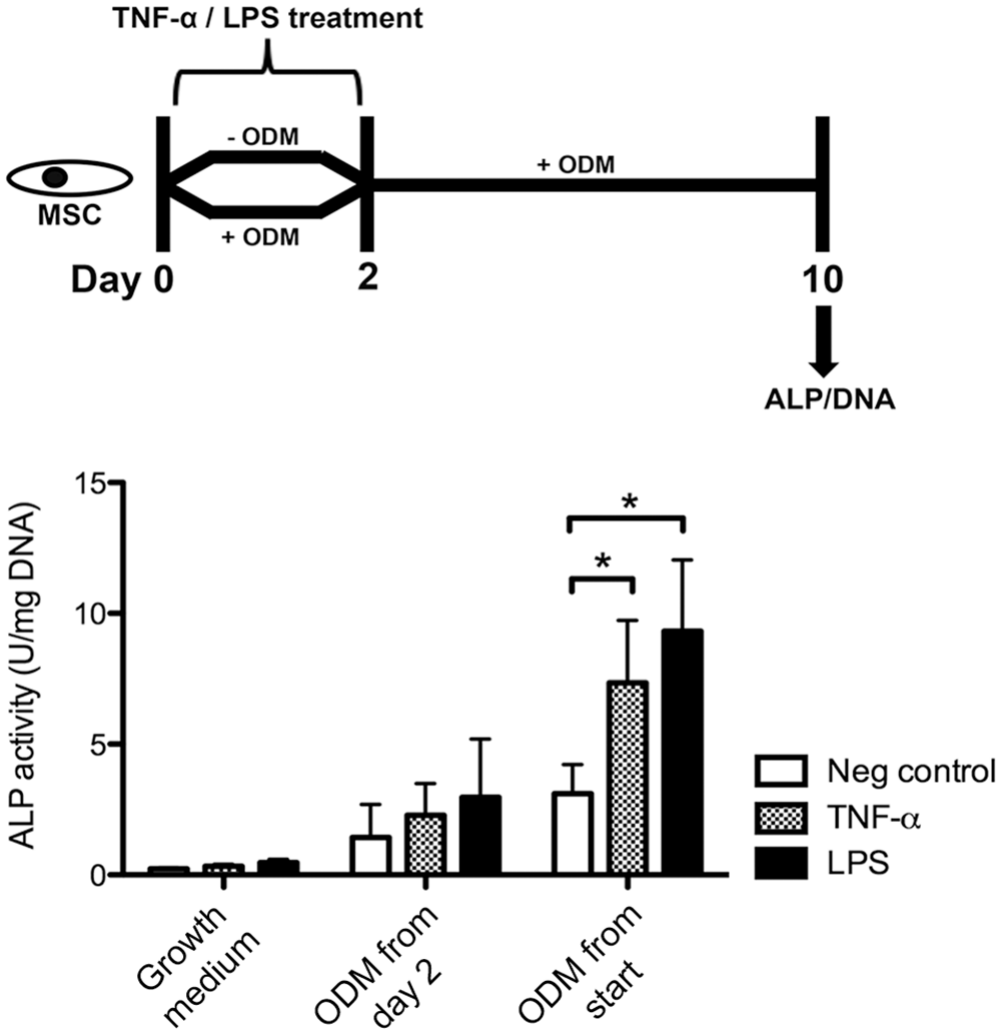
ALP expression by MSCs after short TNF-α or LPS treatment. Cells were exposed to TNF-α (50 ng/mL) or LPS (5 μg/mL) for 2 days, after which the mediators were withdrawn. At day 10, ALP activity levels were measured and normalized for DNA. Data represent the means ± SD (*n* = 4). *P<0.05 compared to untreated cells cultured in the same medium. See S2C Fig for all experimental conditions.

Next, the effect of a brief exposure of these mediators on osteogenesis was studied. This would elucidate if short stimulation of MSCs was sufficient to achieve an osteogenic response, similar to the conditions *in vivo* [27]. Cells were only exposed to the mediators during the initial 2 days (Fig 2). Osteogenic medium was added after the initial 2 days or from the start of the experiment, while growth medium served as a negative control. ALP activity in cultures was measured at day 10.

**Fig 3:**
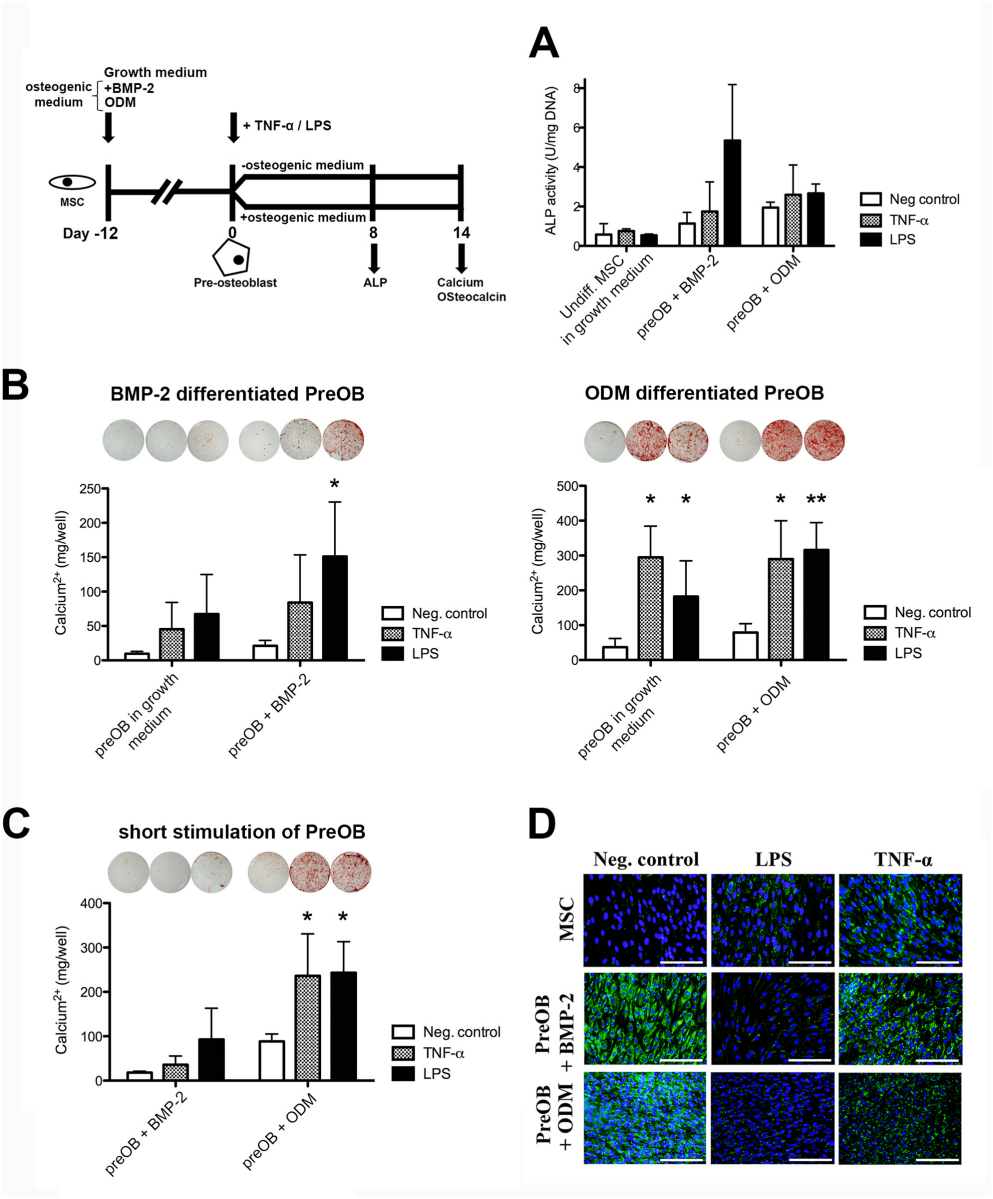
Late osteogenic differentiation in pre-osteoblasts. A. Pre-osteoblasts were exposed to TNF-α (50 ng/mL) or LPS (5 μg/mL). The ALP activity was measured after 8 days and normalized for DNA content. The total calcium deposition was quantified at day 14 after continuous (B) and short (C) stimulation with TNF-α (5 ng/mL) or LPS (0.5 μg/mL). D. Cells were exposed to LPS (0.5 μg/mL) or TNF-α (5 ng/mL) from days 12 to 26 of culture. Immunocytochemical staining for intracellular osteocalcin was performed as a marker of osteogenic differentiation. Osteocalcin and Hoechst are shown in green and blue, respectively (representative for 2 donors). Scale bar: 500 μm. See S4 Fig for a dose response. Data represent the means ± SD (*n* = 4). *P<0.05/**P<0.005 compared to untreated cells cultured in the same medium.

To study the effects of inflammatory signals on osteogenic-committed cells, MSCs were pre-differentiated for 12 days in osteogenic medium. This yields cells that are committed to the osteogenic lineage but not yet undergoing middle or end-stage osteoblastic differentiation [28]. From day 12, these pre-osteoblasts received TNF-α or LPS. This was done in the presence or absence of the osteogenic stimulus they had been exposed to (Fig 3). ALP activity was measured at day 20. At day 26, quantification of mineralization was done using xylenol orange fluorescence staining or Alizarin Red S staining. Furthermore, osteocalcin immunocytochemistry was performed, as this marker is only expressed in osteoblasts/cytes during mineralization [26,29].

### Ethics Statement

Bone marrow was obtained from patients at our institute who had given written informed consent, according to the guidelines of the local medical ethical committee (University Medical Center Utrecht). The ethical committee approved all experiments.

### Human MSC isolation and expansion

MSCs were isolated from bone marrow obtained from patients undergoing arthroplasty at our institute. Male and female donors of different ages were included to ascertain that findings were not unique to a specific source. The mononuclear cell fraction was isolated by centrifugation on Ficoll-paque and plated in expansion medium: growth medium [α-MEM (Invitrogen, Carlsbad, CA, USA), 10% (v/v) heat-inactivated fetal bovine serum (Cambrex, East Rutherford, NJ, USA), 100 units/mL penicillin/streptomycin (Invitrogen)] supplemented with 1 ng/mL basic fibroblast growth factor (R&D Systems, Minneapolis, MN, USA). The cultures were washed after three days to remove non-adherent cells. Cells were always replated below 70% confluency and used between passages 3 and 7. Culture was performed at 37°C in a humidified atmosphere containing 5% C0_2_. The cells from different donors were never pooled.

The multipotency of MSCs using this isolation method has been established previously by standard differentiation assays along osteogenic, adipogenic and chondrogenic lineages [30]. To confirm their phenotype, cells were characterized for the expression of specific surface antigens defining hMSCs [31]. As such, >95% of cells were negative for CD14 and CD45, and >99% of cells were negative for CD19 and CD34. In addition, >95% were positive for CD73, CD105 and CD90 (S1 Fig).

### Osteogenic differentiation assay

MSCs were plated in flat-bottom plates in triplicates at 6,000 or 1,000 cells/cm^2^ for early and late analyses, respectively. Cells were grown until confluency and then exposed to different experimental conditions. For osteogenic differentiation, growth medium (see above) was supplemented with 10 mM of β-glycerophosphate (Sigma-Aldrich) and rhBMP-2 (750 ng/mL, InductOS, Wyeth/Pfizer, New York, NY, USA). In addition, osteogenic differentiation medium (ODM) was used, consisting of 10 mM of β-glycerophosphate and 10 nM dexamethasone/0.2 mM L-ascorbic acid 2-phosphate (Sigma-Aldrich). Medium was changed every 3–4 days.

### Alkaline phosphatase activity

For ALP staining, cells were fixed in 4% (w/v) paraformaldehyde and permeabilized in 0.2% (v/v) Triton X-100 in phosphate buffered saline (PBS). The cell monolayer was incubated for 1 h with the Fuchsin and Chromogen-Substrate system (Dako) and then examined by light microscopy.

For quantitative ALP determination, cells were lysed in 0.2% (v/v) Triton X-100 in PBS for 30 min. ALP activity was measured by conversion of the p-nitrophenyl phosphate Liquid Substrate System (Sigma-Aldrich). The absorbance was measured at 405 nm and corrected at 655 nm (Bio-rad, Hercules, CA, USA). Values were normalized to a standard ALP measurement using serial dilutions of calf intestinal ALP (Sigma-Aldrich) in 0.2% (v/v) Triton X-100 in PBS.

The same cell lysate used to measure ALP was stored at -80°C and subsequently used to determine the DNA content with the Quant-It PicoGreen kit (Invitrogen) according to the manufacturer’s instructions.

### Calcium deposition

For qualitative assessment of matrix mineralization, the cell monolayer was fixed in 4% (w/v) paraformaldehyde, stained for 10 minutes with 2% (w/v) Alizarin red S solution (pH 4.2, Sigma-Aldrich) and examined by light microscopy. To quantify the calcium deposition, samples were incubated with 0.2% (w/v) Alizarin Red S for 60 minutes. Subsequently 10% cetylpyridinium was added for 60 minutes to extract the calcium-bound Alizarin. Absorbance was measured at 595 nm and corrected at 655 nm.

To quantify the amount of calcium present per cell, the calcium-chelating fluorochrome xylenol orange was used. Samples were fixed in 4% (w/v) paraformaldehyde and a solution of 40 μM xylenol orange (Sigma-Aldrich) was added overnight at 37°C in the dark. Prior to fluorescence imaging, cell nuclei were stained with Hoechst (2 μg/mL, bisBenzimide H 33258, Sigma-Aldrich) for 30 min. A total of 25 fluorescence images were collected for each individual well using the ArrayScan XTI (Thermo Scientific, Waltham, MA, USA). Masks were applied around cell nuclei with Cellomic VHS software (Thermo Scientific). The mean fluorescence signal in the xylenol orange channel was determined per nucleus. A threshold value was set to discriminate between calcium-positive and negative cells.

### Osteocalcin immunocytochemistry

Cell monolayers were cultured on glass chamber slides (Lab-Tek, Sigma-Aldrich) and fixed for 15 min in 80% (v/v) methanol. The cell membranes were permeabilized for 10 min by incubation with 0.2% (v/v) Triton X-100 in PBS. After a blocking step of 30 min with 5% (v/v) bovine serum albumin/PBS, samples were incubated overnight at 4°C with 10 μg/mL mouse monoclonal antibody recognizing human osteocalcin (clone OCG4, cat num ALX-804-537, ID AB_2064910, Enzo Life Sciences, Farmingdale, NY). The monoclonal mouse IgG1 antibody (X0931, Dako) was used as isotype-matched control at the same concentration. This was followed by incubation with 10 μg/mL goat-anti-mouse polycloncal antibody conjugated to Alexa Fluor 488 (cat num A-11001, ID AB_141367, Invitrogen). Samples were finally enclosed with VectaShield containing DAPI counterstain (Vector Laboratories, Burlingame, CA,) and evaluated on a fluorescence microscope (Olympus BX51; Olympus DP70, Olympus, Shinjuku, Tokyo, Japan).

### Statistical analysis

All data are expressed as the mean ± standard deviation (SD). Results for individual donors were based on triplicate measurements. Differences between the groups with TNF-α/LPS were considered significant versus the groups without TNF-α/LPS in the same medium when P<0.05 using one-way ANOVA with Bonferroni post-hoc analysis.

## Results

### Early osteogenic differentiation of MSCs

Human MSCs were subjected to proinflammatory stimuli during their early osteogenic differentiation. To examine the mechanism by which these stimuli are involved in lineage determination, we made a distinction between ODM or osteogenic medium supplemented with BMP-2. TNF-α or LPS did not affect the ALP activity in undifferentiated MSCs. Together with an osteogenic inducer, TNF-α or LPS significantly enhanced the ALP activity in MSCs (Fig 1A). In ODM, stimulation with TNF-α (5 ng/mL) or LPS (0.5 μg/mL) increased the ALP activity 3.5-fold versus the group without mediator. In BMP-2 differentiating cells, this induction was 1.5 to 2-fold, respectively. These observations suggest that proinflammatory signals alone do not induce MSC osteogenic lineage commitment but may contribute to the early osteogenic differentiation of hMSCs together with an osteogenic stimulus.

In addition to the early ALP activity, calcium deposition was measured as a late indicator of osteogenic differentiation. We detected limited calcium nodules in the absence of an inflammatory mediator after 22 days. Calcium deposition however markedly increased in a dose-dependent way in the presence of an inflammatory signal (Fig 1B and S2B Fig). As for ALP activity, we observed the highest calcium deposition levels at 5 ng/mL TNF-α and 0.5 μg/mL LPS. For higher concentrations, the amount of calcium declined (S2B Fig). Persistent exposure of MSCs to TNF-α or LPS for 22 days did not induce any calcium deposition by cells in growth medium. To elucidate if inflammatory factors direct the commitment of MSCs specifically to the osteogenic lineage, MSCs were treated with the mediators in adipogenic differentiation medium. Although TNF-α or LPS tended to have both inhibitory and stimulatory effects on the adipogenic differentiation of MSCs respectively, the effect sizes were moderate compared to changes in ALP (S3 Fig). These data collectively show that these inflammatory factors together act with osteogenic inducers to potently stimulate osteogenesis in hMSCs. This is independent of the osteogenic stimuli used, i.e. BMP-2 or dexamethasone/ascorbic acid in ODM.

### ALP activity by MSCs after short exposure

To examine at which stage of lineage commitment proinflammatory stimuli mediate their effect, MSCs were exposed to the mediators during the initial two days only (Fig 2). We found that short TNF-α or LPS treatment significantly increased the ALP activity in differentiating cells, but only if co-delivered with BMP-2 or ODM from the start of culture. The responses were more pronounced in ODM than in BMP-2 differentiating cells (S2C Fig). Although higher concentrations of these mediators were needed to induce this response during brief exposure, i.e. 50 ng/mL TNF-α and 5 μg/mL LPS, the effect sizes were similar compared to their continuous exposure. These observations suggest that osteogenic commitment primes MSCs for their responsiveness to inflammatory signals.

### Late osteogenic differentiation in pre-osteoblasts

To determine the role of proinflammatory stimuli in late osteoblast differentiation, we performed a series of experiments on pre-osteoblasts. Similar to the MSCs, we cultured pre-osteoblasts in growth medium or in osteogenic medium. TNF-α or LPS treatment only moderately enhanced the ALP activity in pre-osteoblasts (Fig 3A). We found the highest ALP activities following LPS treatment in BMP-2 pre-cultured cells. In BMP-2 pre-treated cells, an inhibitive action was observed for high TNF-α concentrations (S4A Fig).

As a decline in ALP activity can be a sign of end-stage osteogenic differentiation at this time point [28], we studied the matrix mineralization in parallel. TNF-α or LPS treatment of pre-osteoblasts increased the amount of deposited calcium (Fig 3B). This was more pronounced for pre-osteoblasts obtained in ODM. Signficant increases in the calcium deposition by these cells were found after treatment with the proinflammatory mediators, even in growth medium (Fig 3B, right panel). In addition, short stimulation of pre-osteoblasts with TNF-α or LPS was sufficient to induce a strong osteogenic response when pre-osteoblasts were cultured in ODM (Fig 3C). Quantification of the amount of calcium per cell by xylenol orange staining demonstrated that the observed increases in matrix mineralization were not clouded by changes in cell proliferation (S4B Fig). The highest matrix mineralization was seen at 5 or 50 ng/mL TNF-α and 0.5 μg/mL LPS (S4B and S4C Fig). Taken together, these data suggest that inflammatory stimuli not only promote the early osteogenic differentiation, but also the late osteoblast differentiation. Furthermore, the strong osteogenic response to the mediators by pre-osteoblasts in the absence of BMP-2 or ODM strengthens the observation that osteogenic commitment primes hMSCs for their responsiveness to inflammatory signals.

We furthermore performed an immunocytochemical staining for osteocalcin as this marker is only expressed by cells committed to the osteogenic lineage. We observed an increased expression of osteocalcin in MSCs cultured in growth medium following treatment with an inflammatory stimulus from day 12 to day 26 (Fig 3D, upper panel). This is in line with the finding that TNF-α or LPS can induce matrix mineralization by MSCs cultured in growth medium for a long period (S4B Fig). Although BMP-2 and ODM culture both induced intracellular osteocalcin expression (Fig 3D, left panel), we found an inhibitory effect of inflammatory stimuli on their osteocalcin expression (Fig 3D, middle and right panels). This was most pronounced for LPS, where we observed almost complete loss of staining. Our data agree with that of others, showing that the osteocalcin expression in MSCs does not predict their matrix mineralization *in vitro* [19].

## Discussion

In this study, we showed that the proinflammatory agents TNF-α and LPS potently enhanced BMP-2 and ODM-induced osteogenic differentiation of human bone marrow MSCs. We characterized the biological mineralization *in vitro* as the main parameter to assess osteogenic differentiation. The ALP activity in MSCs was also quantified to confirm these results, ruling out that possibility of dystrophic mineralization in the cultures. Early ALP activity was preferred over the quantification of osteogenic markers on the RNA level, as it may be more predictive for the *in vivo* bone-forming capacity of human MSCs [32]. Pro-osteogenic effects of these factors alone were only observed in pre-osteoblasts, suggesting that these mediators only stimulated osteogenesis after lineage commitment of bone progenitor cells. In therapies aimed at bone healing and regeneration, the co-delivery of inflammatory mediators may therefore potentiate the effect of bone-promoting factors *in vivo* similar to the early inflammatory response after injury. Alternatively, osteogenic priming of MSCs may be a strategy to increase the efficacy of inflammatory factors in bone regeneration strategies.

Thus far there have been conflicting reports on the effects of TNF-α. Many studies using rodent cells found an inhibitive action of TNF-α on MSC osteogenic differentiation [20,33]. As the few studies using human MSCs as starting cells have yielded comparable results as described here [17,34], species-specific differences likely exist in the action of TNF-α on MSC osteogenic differentiation [35]. Our results on MSCs are furthermore in line with the findings of those who demonstrate stimulatory effects of LPS on the matrix mineralization in human bone marrow- and adipose tissue-derived MSCs [23,36].

We further aimed to investigate timing effects of inflammatory stimuli during very initial lineage commitment. We found similar effects following brief exposure to the mediators, although a 10-fold higher concentration was necessary to achieve this effect. Furthermore, early effects were only observed when MSCs were simultaneously treated with an osteogenic stimulus. To our knowledge, only a single study has obtained results that mirrored the *in vivo* response to TNF-α [18], where brief signaling stimulates bone regeneration and prolonged signaling has destructive effects on bone [6]. These experiments were however performed on primary osteoblasts from bone digests and different timing effects may exist for early MSC osteogenic differentiation.

We further show that the pre-differentiation of MSCs further potentiated the effects of the inflammatory stimuli on matrix mineralization. Interestingly, TNF-α or LPS mediated a stimulatory effect on pre-osteoblast differentiation even without an osteogenic stimulus (Fig 3). This supports the concept that inflammatory stimuli only affect the differentiation of cells committed to the osteogenic lineage. As an exception, inflammatory stimuli also induced an osteoblast phenotype in cells never exposed to an osteogenic supplement. This was demonstrated by a limited matrix mineralization (S4B Fig) and elevated intracellular osteocalcin levels (Fig 3D, upper panel) in growth medium. It has been reported before that MSCs exhibit basal signs of osteogenic differentiation in growth medium when high cell densities are reached [26].

Interestingly, the present data suggest that proinflammatory signals may target downstream regulators of osteogenesis together with osteogenic inducers in bone progenitor cells. Moreover, this action is synergistic, as the effect of TNF-α or LPS with an osteogenic stimulus is larger than the sum of their individual effects. Indeed, some members of the TGF-β superfamily, including BMP-2, are locally expressed immediately after injury during the inflammatory phase of healing [37]. A cross talk is therefore thought to exist between signaling systems from growth factors and inflammatory mediators to recruit MSCs and guide their differentiation. Future studies should further elucidate the molecular mechanisms by which proinflammatory signals act in synergy with BMP-2 or dexamethasone/ascorbic acid to drive their differentiation. We compared the responses to the inflammatory stimuli for different-acting osteogenic inducers. Dexamethasone is thought to antagonize signalling by inflammatory cytokines, possibly masking their true effects [6]. Our report is the first to use BMP-2 as an alternative to dexamethasone to induce osteogenesis in the context of inflammatory mediators. The use of BMP-2 mimics the physiological bone-healing environment and may therefore better simulate the conditions *in vivo* [38]. The outcomes in our experiments were the same following induction with BMP-2 or the synthetic glucocorticoid, even though dexamethasone had a more pronounced effect on the ALP activity in our MSC cultures. The different effects of dexamethasone and BMP-2 on ALP activity in hMSCs *in vitro* have been reported before [39]. Several lines of evidence point uniquely to the fact that their osteogenic effects are mediated by different signaling pathways. BMP-2 induced differentiation primarily involves the Smad proteins. Pathways involving mitogen-activated protein (MAP) kinases, PI3-K, Wnt and NF-κB can further interfere with Smad signaling or induce responses independent of Smads [40–42]. Alternatively, dexamethasone-induced differentiation occurs entirely independent of Smads [28,41,43]. Of the many signaling pathways, modulation of the nuclear factor kappa B (NF-κB) activity by inflammatory mediators is the most likely mechanism by which they exert an effect on the osteogenic differentiation of bone cells. Indeed, the activity of NF-κB is not only enhanced by TNF-α or LPS, but by a wide range of cell harming stimuli [17,22]. Moreover, NF-κB signaling is involved in osteoblast differentiation, demonstrated by the fact that overactivation of NF-κB results in enhanced BMP-2, Runx2 and Osx expression in hMSCs [17]. Importantly, one of the anti-inflammatory mechanisms of dexamethasone is the inactivation of NF-κB in almost all cell types. In MSCs, dexamethasone may therefore interfere with TNF-α/LPS induced signaling [6,44]. In none of the experiments did we observe such antagonizing effects of TNF-α or LPS with dexamethasone. On the contrary, the combination of ODM and a proinflammatory mediator induced a synergistic response. Possibly, anti-inflammatory effects of dexamethasone are achieved at different target cells and only for higher concentrations, ruling out its interfering effects in our osteogenic differentiation assay [44].

In conclusion, the present study shows that TNF-α and LPS directly enhance the differentiation of human bone marrow MSCs, in particular after osteogenic commitment. Moreover, proinflammatory signals likely interplay with common downstream regulators of osteogenesis in bone cells, as we observed a similar outcome for the differentially acting osteogenic inducers BMP-2 and dexamethasone.

## Supporting Information

S1 Fig Characterization of MSCs by flow cytometry. MSCs were stained with the fluorochrome-conjugated antibodies listed under each graph (red histograms), or with the isotype controls for the fluorophore (grey histograms). Cells were incubated for 30 min at 4°C with human FcR blocking reagent (Miltenyi Biotec) and the following monoclonal mouse-anti-human antibodies: CD45 (cat num 560975, ID AB_2033960, BD Biosciences), CD14 (cat num R08641, ID AB_579551, Dako), CD19 (cat num 130-091-328, ID AB_244222, Miltenyi), CD34 (cat num 555821, ID AB_396150, BD), CD73 (cat num 550257, ID AB-393561,BD), CD90 (cat num 328118, ID AB_2303335, Biolegend) and CD105 (cat num FAB10971F, ID AB-356989, R&D Systems). Cell fluorescence was measured in viable cells using a BD FACSCanto II flow cytometer (BD). SytoxBlue (Invitrogen) was used for exclusion of dead cells. Cells were positive for CD90, CD105, CD14b, and CD73, but negative for CD14, CD45, CD19, and CD34.
(TIF)

S2 Fig Stimulation of early MSC osteogenesis by TNF-α/LPS. MSCs were cultured in osteogenic medium consisting of BMP-2 or ODM. Growth medium served as a negative control for osteogenic differentiation. Cells were continuously exposed to TNF-α or LPS. A. ALP activity in MSCs was measured after 10 days and normalized for DNA content (*n* = 6). B. At day 22, Alizarin Red S staining was performed to demonstrate matrix mineralization. Scale bar: 500 μm. C. MSCs were exposed to TNF-α or LPS for 2 days, after which the mediators were withdrawn. Early TNF-α/LPS treatment was performed in the absence (left panel) or presence (right panel) of osteogenic medium. At day 10, ALP activity levels were measured and normalized for DNA content (*n* = 4). Data represent the means ± SD. * P<0.05 versus the group without TNF-α/LPS in the same medium.
(TIF)

S3 Fig Effect of inflammatory mediators on the adipogenic differentiation of MSCs. Cells were seeded and grown until confluency and then differentiated for 10 days using the StemPro Adipogenesis Differentiation Kit (Gibco), according to the manufacturer. Differentiation was performed with or without TNF-α (5 ng/mL) or LPS (0.5 μg/mL). Lipid droplets were stained with an Oil Red O solution and counterstained with hematoxylin. The histogram represents the total area of Oil red O staining as determined by histomorphometry (mean ± SD, *n* = 3). Scale bar: 500 μm.
(TIF)

S4 Fig Effects of proinflammatory mediators on late osteogenic differentiation of pre-osteoblasts. MSCs were cultured in normal growth medium or osteogenic medium consisting of BMP-2 or ODM. Following 12 days of pre-differentiation, cells were exposed to TNF-α and LPS, either in the absence (-osteogenic medium) or presence (+osteogenic medium) of the primary osteogenic stimulus. A. ALP activity levels were measured after 8 additional days of culture and normalized for DNA content (*n* = 2). B. At day 14, calcium expression was measured following the binding of xylenol orange (*n* = 2). C. Alizarin Red S staining confirmed the calcium deposition by cells following TNF-α/LPS treatment, during culture without (-osteogenic medium) or with (+osteogenic medium) an osteogenic stimulus. Scale bar: 500 μm. Data represent the means ± SD.
(TIF)
