# Supplementary figures and images for: Proinflammatory Mediators Enhance the Osteogenesis of Human Mesenchymal Stem Cells after Lineage Commitment
Source: PLoS One. 2015 Jul 15;10(7):e0132781. doi: 10.1371/journal.pone.0132781 (PMC4503569; doi:10.1371/journal.pone.0132781)

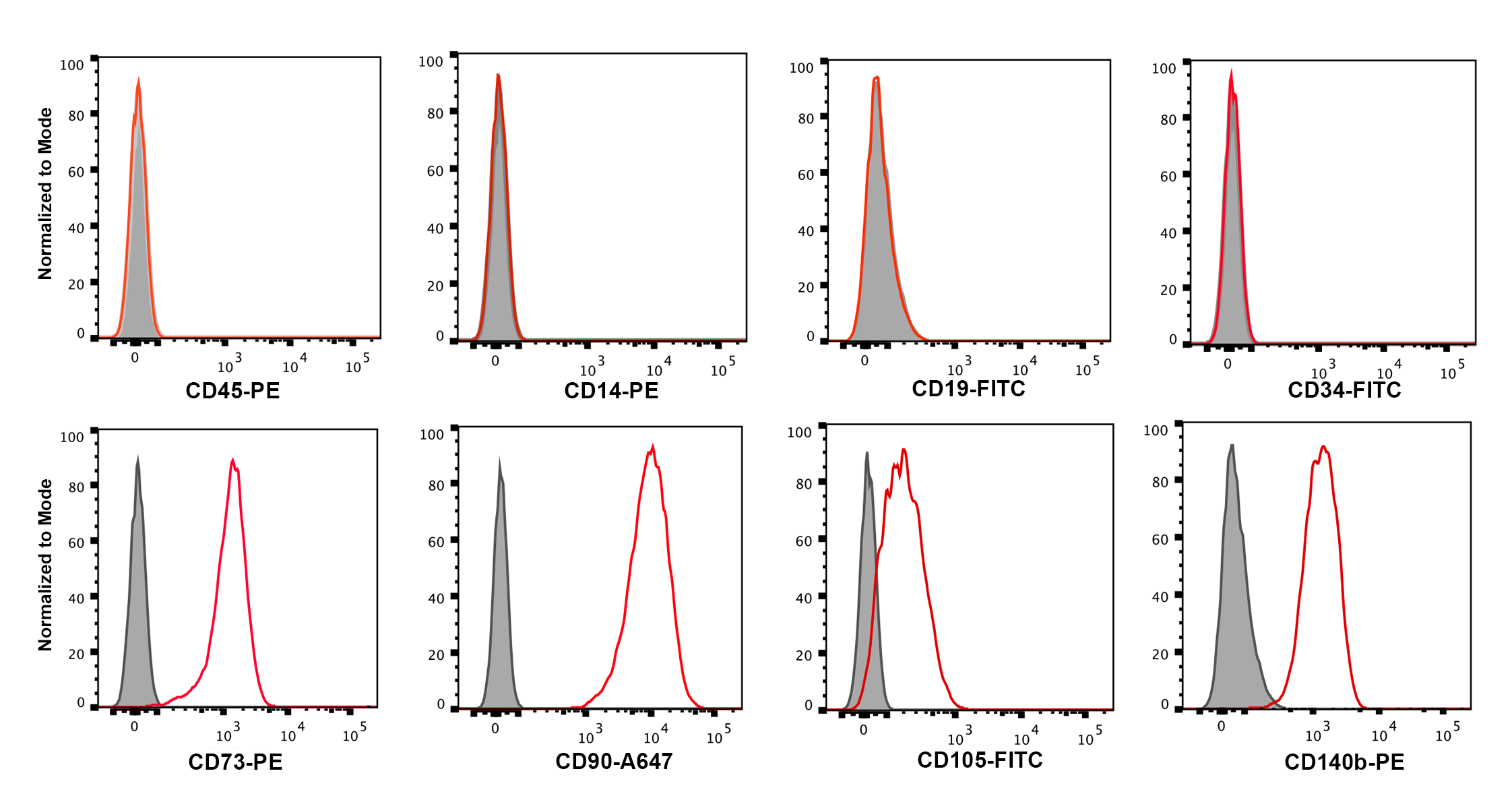

Supplement: S1 Fig — MSCs were stained with the fluorochrome-conjugated antibodies listed under each graph (red histograms), or with the isotype controls for the fluorophore (grey histograms). Cells were incubated for 30 min at 4°C with human FcR blocking reagent (Miltenyi Biotec) and the following monoclonal mouse-anti-human antibodies: CD45 (cat num 560975, ID AB_2033960, BD Biosciences), CD14 (cat num R08641, ID AB_579551, Dako), CD19 (cat num 130-091-328, ID AB_244222, Miltenyi), CD34 (cat num 555821, ID AB_396150, BD), CD73 (cat num 550257, ID AB-393561,BD), CD90 (cat num 328118, ID AB_2303335, Biolegend) and CD105 (cat num FAB10971F, ID AB-356989, R&D Systems). Cell fluorescence was measured in viable cells using a BD FACSCanto II flow cytometer (BD). SytoxBlue (Invitrogen) was used for exclusion of dead cells. Cells were positive for CD90, CD105, CD14b, and CD73, but negative for CD14, CD45, CD19, and CD34. (TIF) [file pone.0132781.s001.tif]

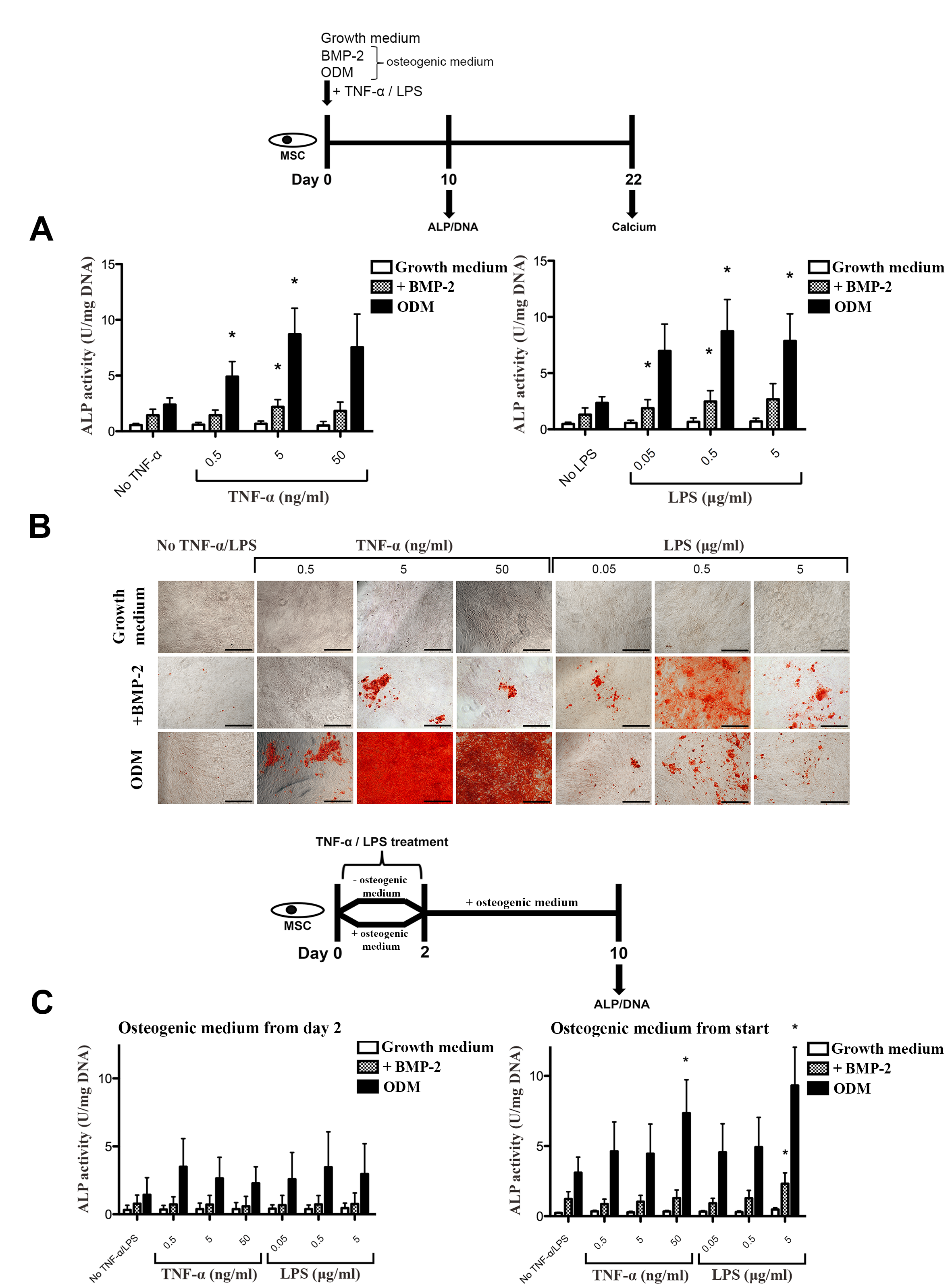

Supplement: S2 Fig — MSCs were cultured in osteogenic medium consisting of BMP-2 or ODM. Growth medium served as a negative control for osteogenic differentiation. Cells were continuously exposed to TNF-α or LPS. A. ALP activity in MSCs was measured after 10 days and normalized for DNA content (n = 6). B. At day 22, Alizarin Red S staining was performed to demonstrate matrix mineralization. Scale bar: 500 μm. C. MSCs were exposed to TNF-α or LPS for 2 days, after which the mediators were withdrawn. Early TNF-α/LPS treatment was performed in the absence (left panel) or presence (right panel) of osteogenic medium. At day 10, ALP activity levels were measured and normalized for DNA content (n = 4). Data represent the means ± SD. * P<0.05 versus the group without TNF-α/LPS in the same medium. (TIF) [file pone.0132781.s002.tif]

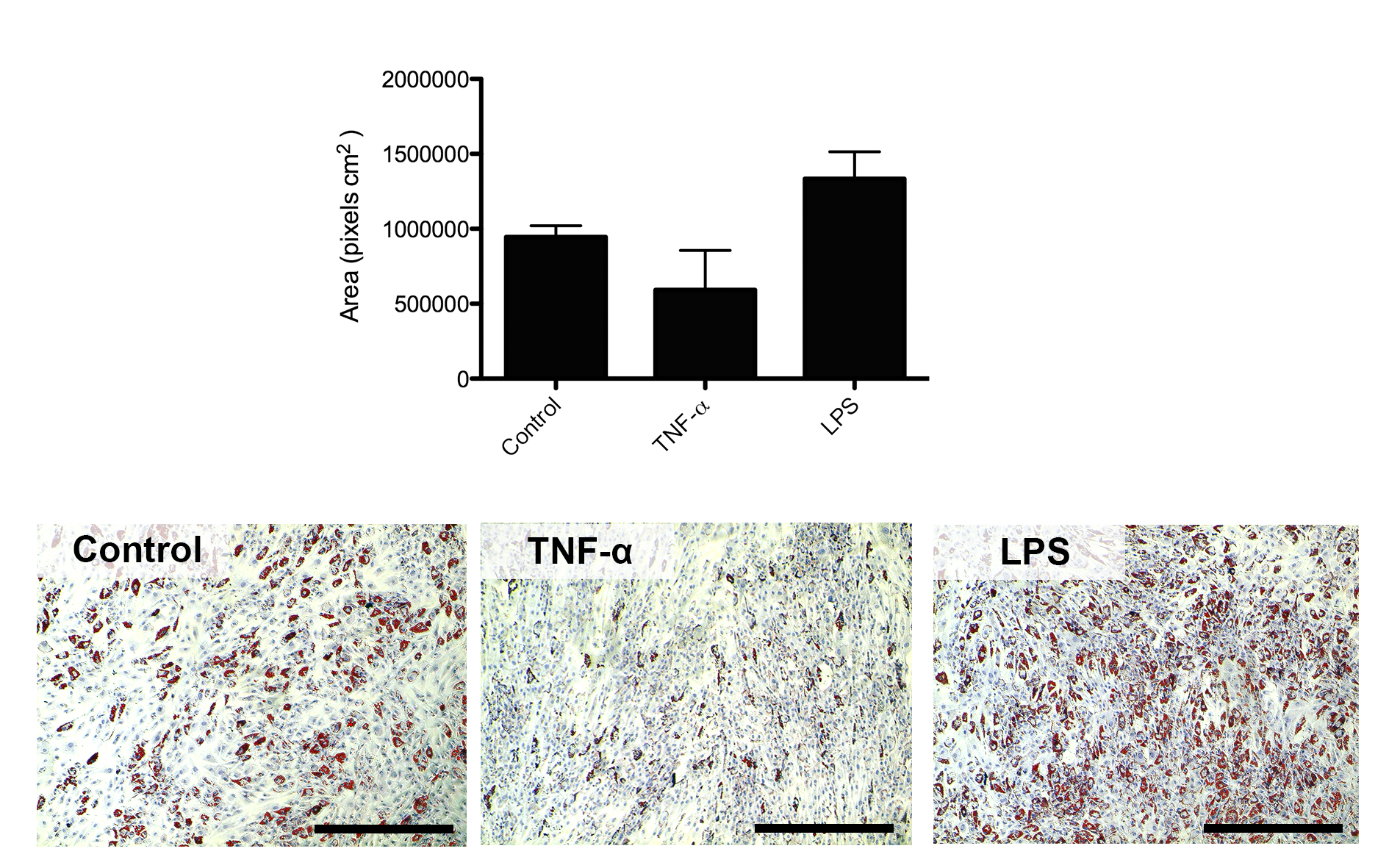

Supplement: S3 Fig — Cells were seeded and grown until confluency and then differentiated for 10 days using the StemPro Adipogenesis Differentiation Kit (Gibco), according to the manufacturer. Differentiation was performed with or without TNF-α (5 ng/mL) or LPS (0.5 μg/mL). Lipid droplets were stained with an Oil Red O solution and counterstained with hematoxylin. The histogram represents the total area of Oil red O staining as determined by histomorphometry (mean ± SD, n = 3). Scale bar: 500 μm. (TIF) [file pone.0132781.s003.tif]

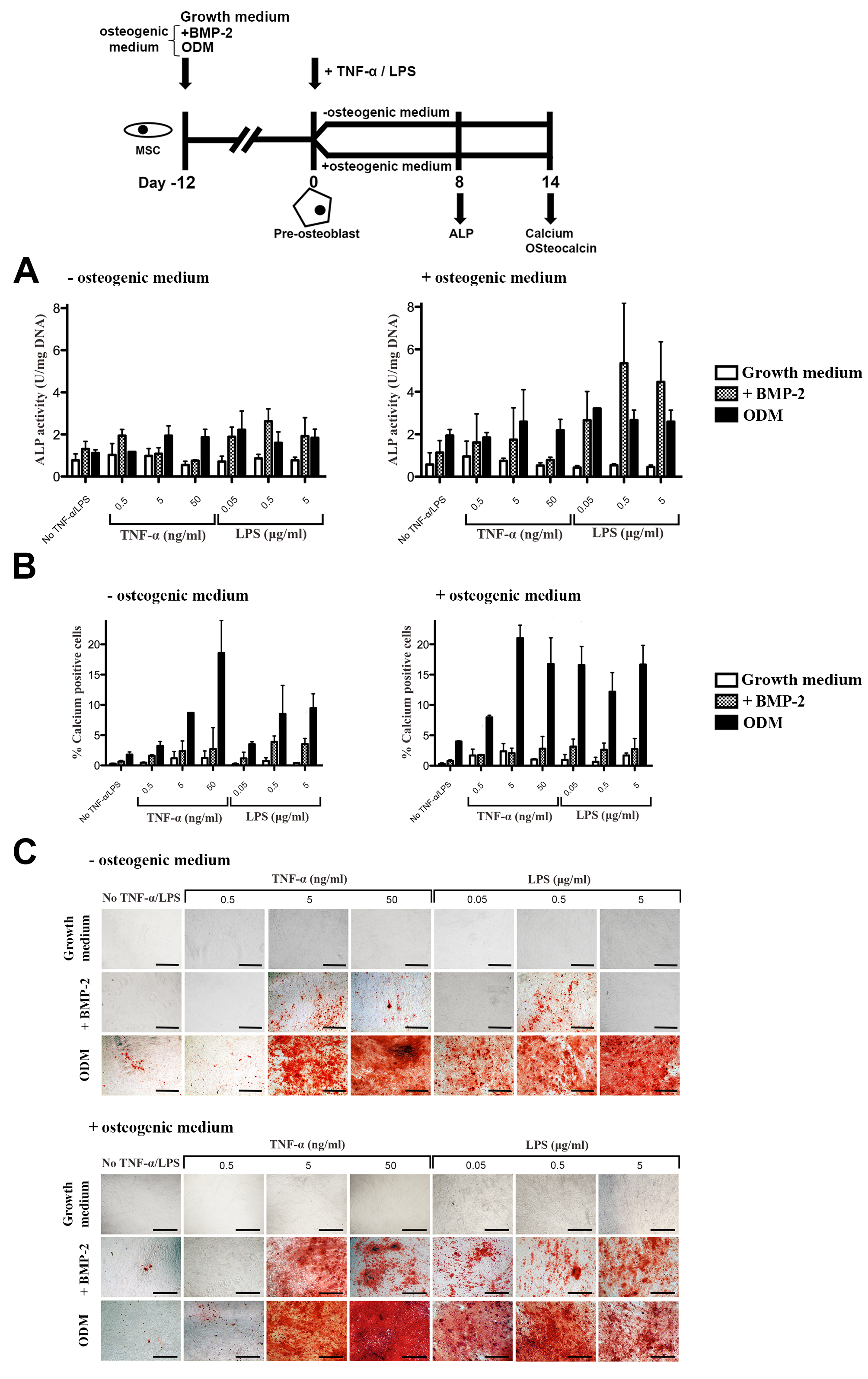

Supplement: S4 Fig — MSCs were cultured in normal growth medium or osteogenic medium consisting of BMP-2 or ODM. Following 12 days of pre-differentiation, cells were exposed to TNF-α and LPS, either in the absence (-osteogenic medium) or presence (+osteogenic medium) of the primary osteogenic stimulus. A. ALP activity levels were measured after 8 additional days of culture and normalized for DNA content (n = 2). B. At day 14, calcium expression was measured following the binding of xylenol orange (n = 2). C. Alizarin Red S staining confirmed the calcium deposition by cells following TNF-α/LPS treatment, during culture without (-osteogenic medium) or with (+osteogenic medium) an osteogenic stimulus. Scale bar: 500 μm. Data represent the means ± SD. (TIF) [file pone.0132781.s004.tif]
